# A case of immunoglobulin A nephropathy treated successfully with tonsillectomy and steroid pulse therapy 20 years after onset

**DOI:** 10.1007/s13730-013-0082-1

**Published:** 2013-07-03

**Authors:** Jumpei Hasegawa, Kei Yamada, Yoshie Kaga, Yasutomo Abe, Mariko Endo, Sachiko Wakai

**Affiliations:** Department of Nephrology, Kidney Center, Ohkubo Hospital, 2-44-1 Kabukicho, Shinjuku-ku, Tokyo, 160-8488 Japan

**Keywords:** Immunoglobulin A nephropathy, Tonsillectomy and steroid pulse therapy, Advanced

## Abstract

A 46-year-old male was found to have proteinuria on a routine medical examination in 1985 at the age of 22 years and was diagnosed with immunoglobulin A (IgA) nephropathy by renal biopsy. He regularly visited a hospital, but 3 years later made the decision to stop. In 2000, his serum creatinine level was 1.3 mg/dl. His renal function then deteriorated, with persistent proteinuria and hematuria, following which he visited our hospital in December 2008. A further renal biopsy was performed. Active and chronic IgA nephropathy was confirmed histologically, with sclerosing lesions also being found. He was treated with three courses of steroid pulse therapy in February and tonsillectomy in April 2009. During the follow-up period at the outpatient clinic, results for proteinuria and hematuria were negative, suggesting that progression of renal impairment had been prevented. The efficacy of tonsillectomy plus steroid pulse therapy for early IgA nephropathy has been demonstrated, and this treatment was effective in our patient 20 years after the onset of the disease.

## Introduction

In 1968, Berger et al. reported that immunoglobulin A (IgA) nephropathy had a favorable prognosis. However, this prognosis was questioned in a report published in 1993, which showed that 10–15 % of patients with IgA nephropathy developed end-stage renal disease (ESRD) within 10 years and 40 % after 20 years [1]. The Ministry of Health, Labor and Welfare and Japanese Society of Nephrology considered the disease a matter of great concern and jointly developed the clinical guidelines for IgA nephropathy in Japan (first published in 1995, the second version in 2002 [2] and the third version in 2011 [3]). These clinical guidelines recommend intervention after careful evaluation of disease activity and treatment. These recommendations are based on a report [4] showing that 20 % of patients recovered spontaneously, with 40 % not progressing to renal impairment.

There are several reports on the efficacy of tonsillectomy and steroid pulse therapy for early stage IgA nephropathy [5, 6], although little is known concerning the best treatment for advanced stage IgA nephropathy.

## Case report

A 43-year old male presented with a history of proteinuria discovered on a routine medical examination in 1985 when he was 22 years old. His medical history was acute pancreatitis at age 17 and hypertension at age 40, and the family history indicated that his nephew had IgA nephropathy. The patient occasionally consumes alcohol, but doesn’t smoke. The patient visited a local doctor and underwent a renal biopsy for suspected active nephritis because of the finding (Table 1) of hematuria 3+ protein from pooled urine 0.78 g/day and a plasma creatinine (Cr) level of 1.16 mg/dl [estimated glomerular filtration rate (eGFR) 71 ml/min/1.73 m^2^]. Pathologic examination revealed IgA nephropathy with partial cellular crescent formation in 1 of the 12 glomeruli, slight mesangial matrix expansion, and mild atrophy of the renal tubule and interstitium. He was followed up without treatment, and the urinary abnormalities resolved spontaneously. He stopped visiting the hospital 3 years after the first visit because of the repeated negative urine analysis and did not visit any other hospital thereafter. He continued to undergo routine medical examinations, which were intermittently positive for microhematuria and proteinuria. His next visit to a medical institution was in 2002 at 38 years of age and revealed urine protein 2+, urine occult blood 1+, plasma Cr 1.13 mg/dl and hypertension. An angiotensin receptor blocker (ARB) was started. After that, he saw a doctor regularly, but hematuria and proteinuria became gradually worse. A relapse of IgA nephropathy was suspected. In March 2009, he was admitted to a hospital for renal biopsy to evaluate the progression of IgA nephropathy.

**Table 1:** The clinical course of laboratory findings

	1985 (22 years old) First renal biopsy	2000 (35 years old) Follow-up of local doctor	2008 (43 years old) Second renal biopsy	2009 (44 years old) Clinical remission 7 months after steroid pulse therapy	2013 (43 years old) The last outpatient visit
Urinary occult blood	3+	–	3+	–	–
Urinary Protein	3+ 0.78 g/day	1+ ND	3+ 0.90 g/gCr	– 0.04 g/gCr	– 0.03 g/gCr
Serum Cr (mg/dl)	1.16	1.30	1.63	1.20	1.40
eGFR (ml/min/1.73 m^2^)	71.0	48.5	38.7	53.0	45.1

The patient's general condition at the time of hospitalization was height 163.0 cm, weight 55.0 kg, body mass index 20.7 kg/m^2^, blood pressure 116/72 mmHg, heart rate 83 beats/min and temperature 36.6 °C. He was lucid with no sign of anemia in the palpebral conjunctiva. No swelling of the throat, abnormal chest auscultation, hard and tender points in the abdominal area or edema of the extremities was observed. Medical examinations (Table 1) revealed urine protein 3+ (0.90 g/day), urine occult blood 3+ (RBC 10–19/HPF) and serum Cr 1.63 mg/dl.

**Fig. 1:**
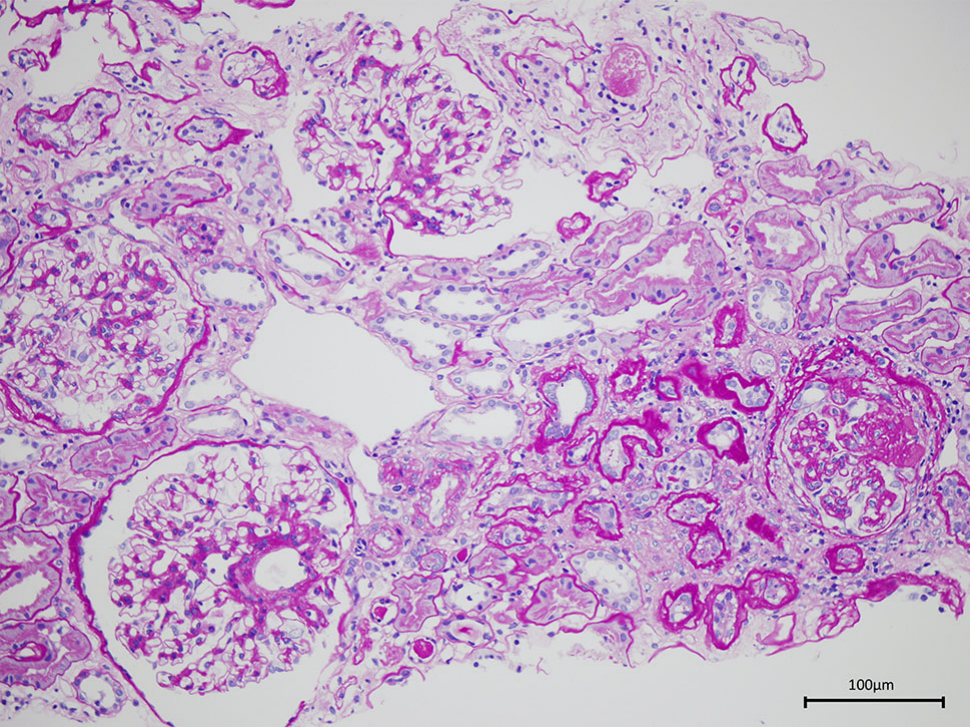
Renal biopsy findings. PAS staining showed moderate tubular atrophy and globally sclerotic glomeruli

Renal biopsy (Fig. 1) showed 4 global scleroses, 3 cellular crescent formations and 3 adhesions in 28 glomeruli; moderate expansion of the mesangial matrix; moderate tubular atrophy with mild cell infiltration in the interstitium; and moderate arteriolosclerosis and thickening of interlobular arteries. Immunostaining of IgA confirmed the diagnosis of active chronic-progressive IgA nephropathy.

**Fig. 2:**
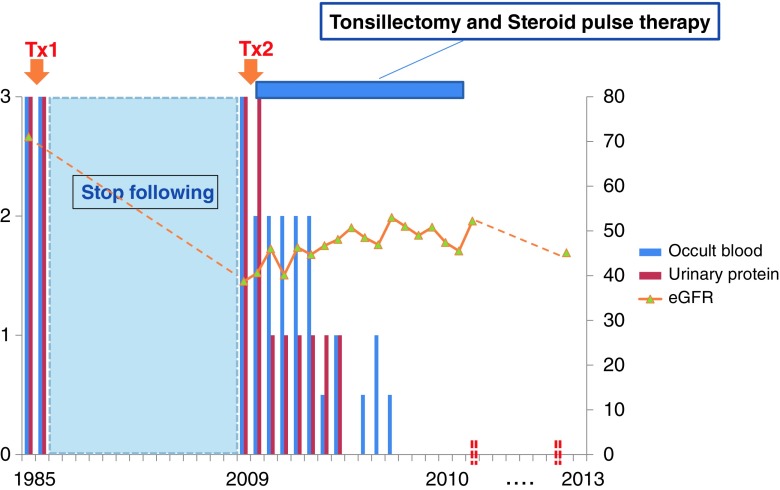
The clinical course

After admission, the patient was treated with tonsillectomy and steroid pulse therapy. Steroid pulse therapy was given as follows: methylprednisolone was administered continuously at a pulsed dose of 500 mg/day for 3 days, followed by oral prednisolone at a dose of 30 mg continuously for 3 weeks. After completion of the third course, oral prednisolone was initiated at a dose of 30 mg/day every other day after treatment and tapered by 5 mg every 2 months. The after-treatment follow-up was continued for 1 year. The patient underwent tonsillectomy after discharge from the hospital (day 75) and was then followed up at the outpatient clinic. He completed oral prednisolone treatment in approximately a year, but continued taking an ARB during the follow-up period. Clinical remission was achieved 7 months after the initiation of therapy (Table 1), and hematuria and proteinuria were no longer observed in this 3-year follow-up period (Fig. 2).

## Discussion

It is well known that patients with IgA nephropathy experience different disease courses. Our patient had a peculiar clinical course. On diagnosis of mild IgA nephropathy and follow-up without treatment, he temporally underwent spontaneous remission, although relapse occurred 14 years later. As a result of his long-term survival with moderate renal failure, the second renal biopsy showed a composite result of more advanced chronic manifestation and active disease with decreased eGFR of 38.7 ml/min.

Since Hotta et al. [7] reported the use of tonsillectomy and steroid pulse therapy in patients with IgA nephropathy in 2001, the therapy has been used extensively in patients with IgA nephropathy mainly in Japan as well as in other countries. As mentioned above, some patients have spontaneous remission; therefore, the criteria for its use have not yet been established.

There are several reports on the efficacy of tonsillectomy and steroid pulse therapy for nonadvanced IgA nephropathy, especially in Japan [5, 6]. Although the indication of tonsillectomy and steroid pulse therapy remains controversial, it is hypothesized that there is a “point of no return” in progressive IgA nephropathy after which progression to end-stage renal disease (ESRD) becomes inevitable. Studies on treatment for advanced IgA nephropathy include a retrospective cohort study by Sato [7] who investigated 70 patients with baseline serum Cr of 1.5 mg/dl or higher, who were assigned to the tonsillectomy and steroid pulse therapy, oral steroid or control groups and followed up for a mean of 5.9 years. The results showed that patients with baseline serum Cr of 1.5–2.0 mg/dl in the tonsillectomy and steroid pulse therapy group had decreased progression of ESRD compared with the other two groups, whereas there was no significant difference in the outcome in patients with baseline plasma Cr of ≥2.0 mg/dl. Komatsu et al. [8] reported similar results, indicating that tonsillectomy and steroid pulse therapy are not indicated in patients with a Cr level above 2.0 mg/dl.

Our patient was considered to have a poor prognosis as he had a long disease duration of 24 years and advanced renal impairment with eGFR of 38.7 ml/min and plasma Cr of 1.41 mg/dl. However, the IgA nephropathy continued to present disease activity; therefore, we performed tonsillectomy and steroid pulse therapy. Remission of the urinary findings was achieved, and progressive deterioration of renal function was successfully prevented. Although a chronic manifestation such as glomerulosclerosis remained, eGFR improved following suppression of acute inflammatory symptoms. Hotta et al. [9] reported that tonsillectomy and steroid pulse therapy were associated with histological improvements such as reduced mesangial proliferation and interstitial fibrosis [10]. Subsequent development of nephrosclerosis may be reduced mainly by management of hypertension and nutritional therapy with protein and sodium restriction preventing hyperfiltration in residual glomeruli.

This case report shows the possibility of the effect of tonsillectomy and steroid pulse therapy for IgA nephropathy with long disease duration and decreased renal function if the activity of IgA nephropathy remains.
